# Role of gene signatures combined with pathology in classification of oropharynx head and neck cancer

**DOI:** 10.1038/s41598-020-66983-x

**Published:** 2020-06-23

**Authors:** Andrew Dhawan, Jacob Scott, Purnima Sundaresan, Michael Veness, Sandro Porceddu, Eric Hau, Adrian L. Harris, Francesca M. Buffa, Harriet E. Gee

**Affiliations:** 1 Department of Oncology, University of Oxford, Oxford, United Kingdom; 2 Translational Hematology and Oncology Research, Cleveland Clinic, Cleveland, Ohio, USA; 3 Crown Princess Mary Cancer Centre, Westmead Hospital, Westmead, New South Wales Australia; 4 Sydney Medical School, C24 - Westmead Hospital, The University of Sydney, Sydney, New South Wales Australia; 5 Princess Alexandra Hospital, Woolloongabba, Queensland Australia; 6 Faculty of Medicine, The University of Queensland, Brisbane, Queensland Australia

**Keywords:** Head and neck cancer, Tumour biomarkers

## Abstract

Treatment personalisation remains an unmet need in oropharynx cancer (OPC). We aimed to determine whether gene expression signatures improved upon clinico-pathological predictors of outcome in OPC. The clinico-pathological predictors, AJCC version 7 (AJCC 7), AJCC 8, and a clinical algorithm, were assessed in 4 public series of OPC (n = 235). Literature review identified 16 mRNA gene expression signatures of radiosensitivity, HPV status, tumour hypoxia, and microsatellite instability. We quality tested signatures using a novel *sigQC* methodology, and added signatures to clinico-pathological variables as predictors of survival, in univariate and multivariate analyses. AJCC 7 Stage was not predictive of recurrence-free survival (RFS) or overall survival (OS). AJCC 8 significantly predicted RFS and OS. Gene signature quality was highly variable. Among HPV-positive cases, signatures for radiosensitivity, hypoxia, and microsatellite instability revealed significant underlying inter-tumour biological heterogeneity, but did not show prognostic significance when adjusted for clinical covariates. Surprisingly, among HPV-negative cases, a gene signature for HPV status was predictive of survival, even after adjustment for clinical covariates. Across the whole series, several gene signatures representing HPV and microsatellite instability remained significant in multivariate analysis. However, quality control and independent validation remain to be performed to add prognostic information above recently improved clinico-pathological variables.

## Introduction

Cancer of the oropharynx (base of tongue, tonsil, and pharynx, OPC), is a debilitating disease. Treatment with combined chemoradiotherapy has a significant impact on acute and long-term quality of life^1^. A remarkable increase in the incidence of OPC related to infection with the human papilloma virus (HPV), has occurred in developed countries^2–4^, with high physical, emotional, and social costs^5^. While HPV+ OPC can be cured with 3 year overall survival greater than 90%^6,7^, survival rates are approximately 60% for non-HPV-associated oropharyngeal cancer (HPV−).

Response to radiation varies markedly in OPC; in general, HPV+ cancers are 2-3x more radiosensitive than HPV- cancers^8^. Multiple mechanisms have been suggested to explain the greater radiosensitivity of HPV+ OPC including retention of functioning p53^9^, defects in DNA repair^10,11^, and others^12,13^. However, a spectrum of radiotherapy response exists, and it is difficult to predict individual tumor radiosensitivity before treatment^14^. Better predicting these differences upfront would enable treatment ‘de-escalation’ for patients with radiosensitive tumours, and more rational treatment ‘escalation’ (e.g. with individualised chemotherapy or immunotherapy) for patients with radioresistant tumours.

Multiple markers of radiosensitivity have been investigated. However, most conventional clinico-pathological markers such as tumour grade, size, nodal burden and stage poorly predict response to radiation^15–18^. In OPC, the best described is immunohistochemistry for p16, a surrogate marker of integration of high-risk HPV into the host genome. p16 status (positive indicating an HPV+ tumour) has very recently been integrated into the latest American Joint Committee on Cancer (AJCC)^19^ and UICC staging systems^20^, and has been used to select patients for clinical trials^21^. However, p16 status does not perfectly predict response to treatment, particularly in patients who have a history of smoking^8^.

Given the limitations in histochemical pathologic markers, several gene expression-based signatures of response to radiotherapy have been described. These include a radiosensitivity index (RSI), which has been used to determine a genome-based model for adjusting radiotherapy dose (GARD)^22^ and several RSIs derived from the radiation response of NCI-60 cell lines^23,24^. However, RSIs have not been adjusted for confounding factors, such as tumour hypoxia, which is known to impact radioresistance. Several other gene expression signatures capture these related biological parameters such as hypoxia^25–27^, immune function^28^, and HPV status. In addition, virus-related cancers may be more sensitive to immunologic checkpoint inhibitors, leading to intense interest in surrogate markers, of which the best described is microsatellite instability (MSI)^29^. MSI has been associated with prognosis^30^ and gene expression signatures have been described^31,32^.

Gene expression signatures are being introduced to the clinic as they become cheaper and easier to obtain. For example, GARD has been proposed as the basis for a prospective, biologically-guided trial of radiotherapy dose de-escalation in head and neck cancer^22^. However, in general, the quality of gene signatures has not been independently validated, and are often poorly reproducible across multiple tumour types, requiring large scale trials for validation^33^.

The purpose of this study was to determine whether currently available gene expression signatures improved upon established and recently-improved clinico-pathological predictors of outcome, to prognosticate more accurately in OPC.

## Materials and Methods

### Clinical datasets

**Figure 1:**
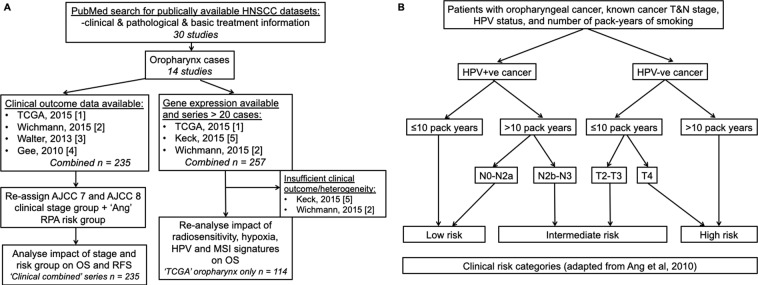
(**A**) Pubmed search strategy, outlining datasets identified in study. (**B**) Risk group staging approach as adapted from Ang *et al*.^8^.

A Pubmed search was undertaken (Fig. 1A, Table 1) for datasets with clinical, pathological, and basic treatment information. We found 235 cases (called ‘*Clinical combined*’) across four series (TCGA(1)^34^; and first authors Wichmann(2)^35^, Walter(3)^36^, and Gee(4)^18^) with sufficient clinical information to assign stages with both AJCC versions 7 and 8. Stage was manually assigned using the parameters in the appropriate manual^19,37^. HPV status was assessed by the methods described by the original authors. Risk group as per Ang *et al*.^8^ was assigned for cases among these four studies where the requisite pathological and smoking information was available. This system, outlined in Fig. 1B, classifies patients by their HPV-status, smoking history, and tumour/nodal status. All TCGA datasets used in this project were accessed through the Broad Institute Firebrowse portal at www.firebrowse.org, and the most updated version is available at https://portal.gdc.cancer.gov/.

**Table 1:** Demographics and clinical characteristics of ‘combined’ cohort (N = 235).

Characteristic	Number (percentage or range)
Median age in years at diagnosis (range)	57 (35–80)
Number female (%)	32 (13.6%)
HPV status:	
Overall HPV positive	116 (49%)
Method of HPV status determination‘:	
HPV RNA positive (RNA seq)	42
HPV DNA (ISH) positive	64
p16 immunohistochemistry (IHC)	10
Negative	98 (46%)
Unknown	11 (5%)
Subsite:	
Base of tongue	39 (16.5%)
Oropharynx*	142 (60.5%)
Tonsil	54 (23%)
Clinical Stage AJCC 7:	
I	10 (4.2%)
II	23 (9.7%)
III	33 (13.9%)
IV	18 (7.6%)
IVa	134 (57%)
IVb	13 (5.5%)
IVc	4 (1.7%)
Clinical Stage AJCC 8:	
I	71 (30%)
II	40 (17%)
III	44 (19%)
IV	9 (4%)
IVa	51 (21.8%)
IVb	4 (2%)
IVc	1 (0.2%)
NA	15 (6%)
Number with more than 10 pack year history (%)	186 (81%)
Median pack years of smokers (IQR)	30 (12–60)
Number of patients receiving radiotherapy (%)	177 (75%)
Number of patients receiving surgery (%)	57 (24%)
Number of patients receiving chemotherapy (%)	110 (47%)
Median follow up (years)	2.34
Number of events (overall survival)	83
Number of events (recurrence free survival)	99

AJCC = American Joint Committee on Cancer.

‘Method of determination as described by authors of original paper. For details of overlap between different methods, if performed, please see Supplementary Table A4.

*Several series – specifically Walter and Wichmann consisted of 100% ‘oropharynx’ cases with no further definition of subsite (Supplementary Table A1).

### Gene expression and signature analyses

While three studies were found with gene expression analyses for >20 cases, two of these series could not be analysed due to systematic bias upon baseline quality control check (Wichmann (2)) or incomplete clinical data (Keck (5)). For analysis of gene expression, cases from TCGA (series 1) head and neck cancers were used^34,38^ (limited to those from oropharynx, tonsil, or base of tongue, Supplementary Table A1). 16 Gene signatures and two single genes were identified by comprehensive literature review and cross checking through reputable databases, such as MSigDB from the Broad Institute (see Supplementary Figure 1 for method, Supplementary Tables A2 and A3 for details). Signatures that were derived on TCGA were excluded from analysis. Only one gene signature (the RSI) was provided with an associated linear model. All others were presumed to act as metagene-based signatures, and median expression of the individual genes was used as the score metric for each of these. For this reason, the expression up and expression down genes of each of these signatures were considered separately, as the median score requires all genes of a signature to be changing in the same direction. mRNA expression data was normalised using the RNA-Seq Expectation Maximization methodology (RSEM). Data were log-transformed by taking log_2_(x + 1) for the RSEM normalised expression level for the mRNA, x. In analyses involving TP53 mutational status, the status for each sample was summarised as a binary variable (1) if the sample had a non-silent mutation in the TP53 gene.

## Statistical methodology

### Gene signature quality control

Signatures with at least two genes present within the TCGA dataset were evaluated for applicability to the study dataset considered prior to use within survival analyses. *sigQC* was implemented for each gene expression signature on this dataset, with quality control summaries in Appendix B. We identified 16 gene expression signatures of variable length and two single genes, as outlined in Supplementary Tables A2 and A3 and Supplementary Figure 1 (flowchart). Each gene signature was summarised into a single score for the purpose of analysis, using the median expression of each of the signature genes in the normalised dataset, and was shown to have strong correlation to other metrics of signature score, and strong variability across the dataset. When used as predictors, these scores were transformed into fractional ranks, as described below. This ensured that signatures were used as metrics for sample ranking across the dataset, and differences in scale or expression of genes did not impact the results of our analysis.

### Univariate survival analysis

Univariate survival analysis was performed using a linear Cox proportional hazards model for the log of the hazard ratio, with the response variable as overall survival, and univariate predictors as previously described^39^. Age greater than or equal to 60 years, HPV-status and smoking, and whether radiotherapy was received were considered as binary variables. Either p16 status or other detection methods for HPV directly as per original series was considered positive.

### Multivariate survival analysis

For each gene signature or single gene predictor, we fit a linear model to a combination of the stage (7th edition), age, smoking status, HPV status, radiotherapy, and gene signature score or expression value. Because not every patient could be restaged to the 8^th^ edition AJCC staging system, staging was used from the 7th edition to retain statistical power. Multivariate survival analysis was performed using the variables as above using Cox proportional hazards estimation for the log of the hazard ratio. The model used consisted of each of the clinical predictor variables as described in the univariate analysis and the scores of a gene signature.

## Results

### Restaging with AJCC 8th edition yields more even distribution of staged cases and improved prognostication

**Figure 2:**
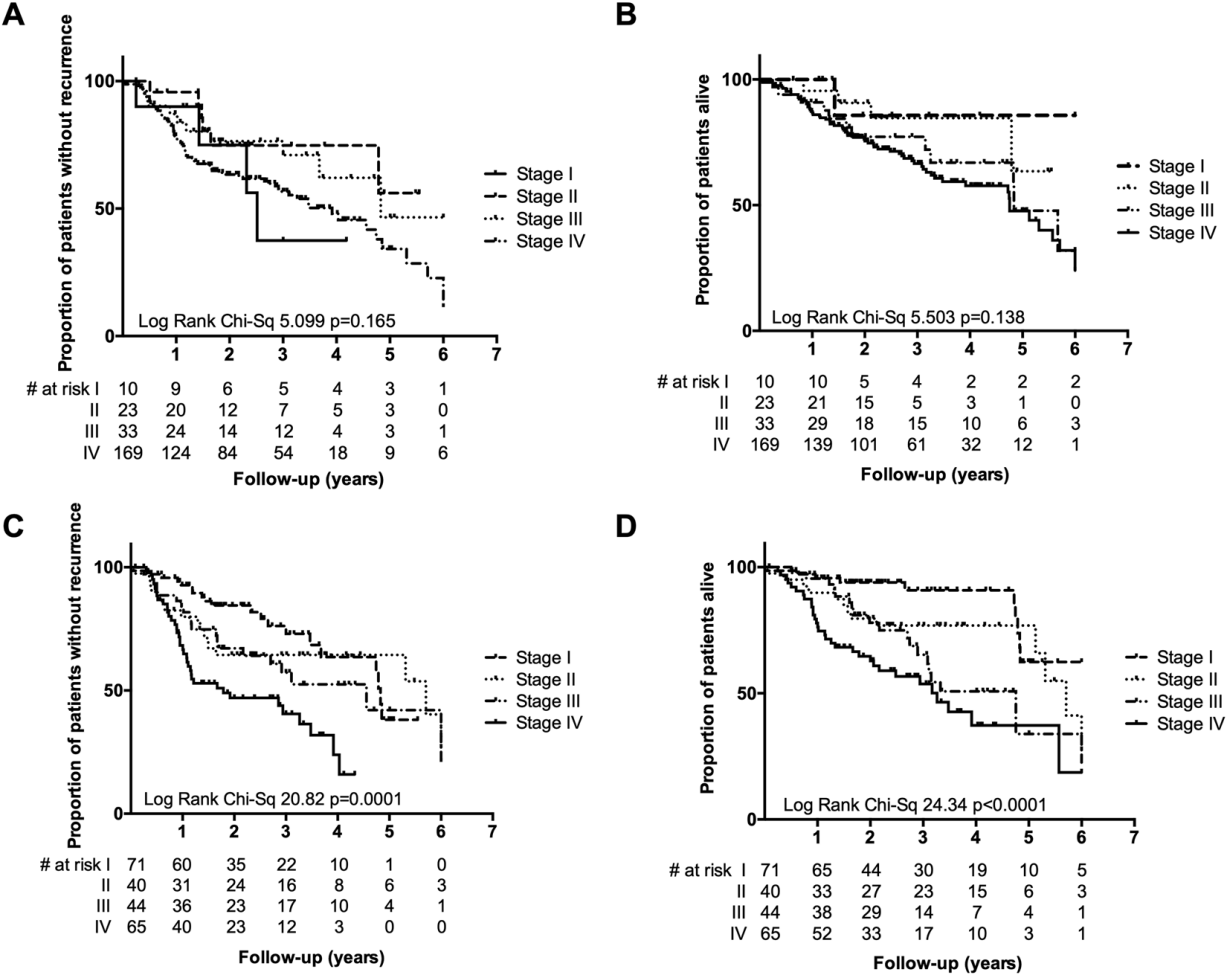
Among patients considered in four combined series of OPC, AJCC 7^th^ edition staging does not significantly stratify for RFS (**A**) or OS (**B**). AJCC 8^th^ edition staging shows statistically significant stratification for RFS (**C**) and OS (**D**). Number at risk is given for each group.

We first examined the utility of a commonly-used staging system. A literature search found four series for which pathological and outcome information was available (Fig. 1A, Table 1 and Supplementary Table A1, n = 235, henceforth called ‘*Clinical Combined’* series). Clinical stage grouping, based on traditional American Joint Committee on Cancer (AJCC) criteria (7th Edition, 2010)^37^, was, as expected, highly skewed towards advanced stage (Stage I = 4%, Stage II = 10%, Stage III = 14%, Stage IV = 72%). AJCC 7 stage did not correlate with RFS (p = 0.188) or OS (p = 0.158) (Fig. 2A,B). The 8th edition of the AJCC staging system incorporates immunohistochemistry for p16 as a surrogate marker of integration of high-risk HPV into host genome in OPC^19^. Individual patient data were used to re-stage across ‘*Clinical Combined’* series. Cases were more evenly distributed across the four stages (Stage I = 30%, Stage II = 17%, Stage III = 19% and Stage IV = 34%), and AJCC 8 significantly predicted response to treatment (p < 0.0001 for both RFS and OS), noting that these patients were treated under the previous staging paradigm (Table 1, Fig. 2C,D).

### Clinically-identified groups based on p16 positivity, smoking status, and T/N stage still do not consistently predict outcome

**Figure 3:**
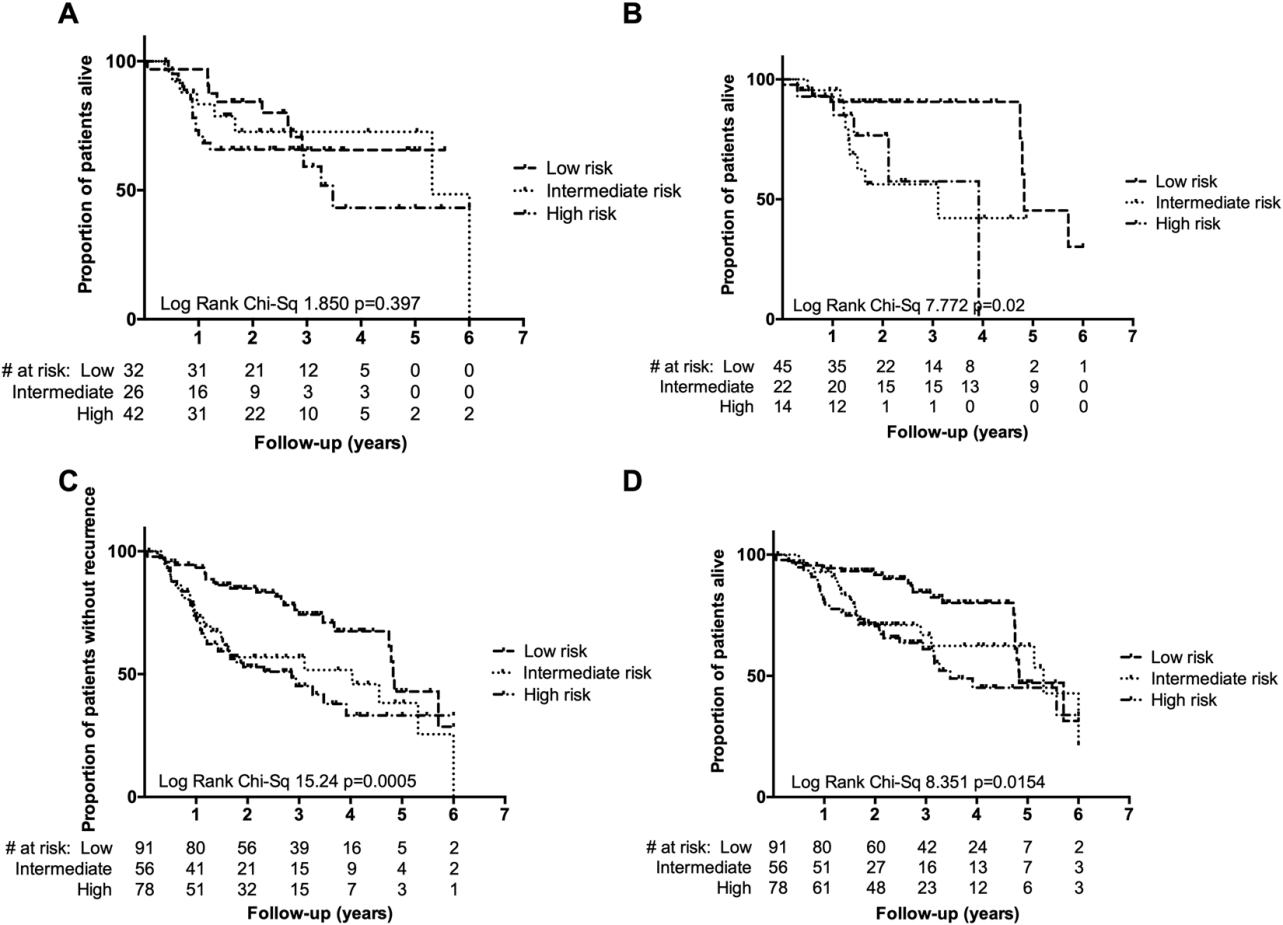
Clinically-identified risk groups do not significantly stratify intermediate and high risk groups of patients when considering OS in the Wichmann series (**A**) and the TCGA series (**B**). These risk groups significantly stratify patients in the combined series with respect to RFS (**C**) and OS (**D**), but again show overlap among intermediate and high risk groups. Number at risk is given for each group.

The three clinical risk groups for OPC identified by Ang and colleagues^8^ found by retrospective recursive partitioning analysis of a large randomised controlled trial are widely used informally in clinical practice (along with the ICON-S staging system, which led to the update to AJCC 8). Patients are classified as low, intermediate or high risk on the basis of a combination of p16 status, smoking status (greater or less than 10 pack years) and T/N stage (Fig. 1B). We tested this clinico-pathological risk assessment in individual series and in the ‘*Clinical combined*’ series. We found that although the three groups were reproducible, differentiation between the intermediate and high-risk groups (Fig. 3) was incomplete, suggesting utility of further biological information.

### Gene signatures show variable quality on TCGA dataset

Given the limitations of clinico-pathological variables in predicting response to radiation, a literature search was performed for gene expression-based signatures of radiosensitivity, hypoxia, HPV status, and microsatellite instability. We identified 16 gene expression signatures of variable length and 2 single genes, as outlined in Supplementary Tables A2 and A3 and Supplementary Figure 1 (flowchart). These were tested on TCGA dataset (as this was the only publicly-available dataset worldwide with all requisite parameters available). The other two series with gene expression data available were excluded due to insufficient clinical information (Keck) and the observation of systematic bias on preliminary *sigQC* analysis (Wichmann). Notably, *sigQC*^40^ acts to check the expression, distribution, and variance of gene signature genes on a given dataset, compared to a set of null controls, to better ascertain the legitimacy of using a dataset/signature combination.

Using the protocol outlined through the R package *sigQC*^40^, a suite of metrics was computed to test the quality of each gene signature’s applicability to the TCGA dataset, revealing a wide range of signature quality. In particular the Kim 2012, Up^24^, Pyeon HPV, Down, Pyeon HPV, Up^41^, and Amundson 2008^23^ signatures were the strongest performers, and the Watanabe MSI, Up^32^ signature was the poorest (Appendix B). Each gene signature had nearly all genes represented in the TCGA dataset, and good variability of these sets of genes was shown (including median coefficient of variation of signature genes within the 25^th^–75^th^ percentiles when compared to all genes, and median standard deviation of signature genes across the samples of the dataset was between the 50^th^–75^th^ percentiles when compared to all genes). *sigQC* thus gave the confidence for the application of these gene signatures on the TCGA dataset, but does not provide a means to assess quality of a signature as a prognostic biomarker, necessitating further analysis.

### Univariate and multivariate analysis identifies prognostic value of gene signatures and TP53 mutation status

**Figure 4:**
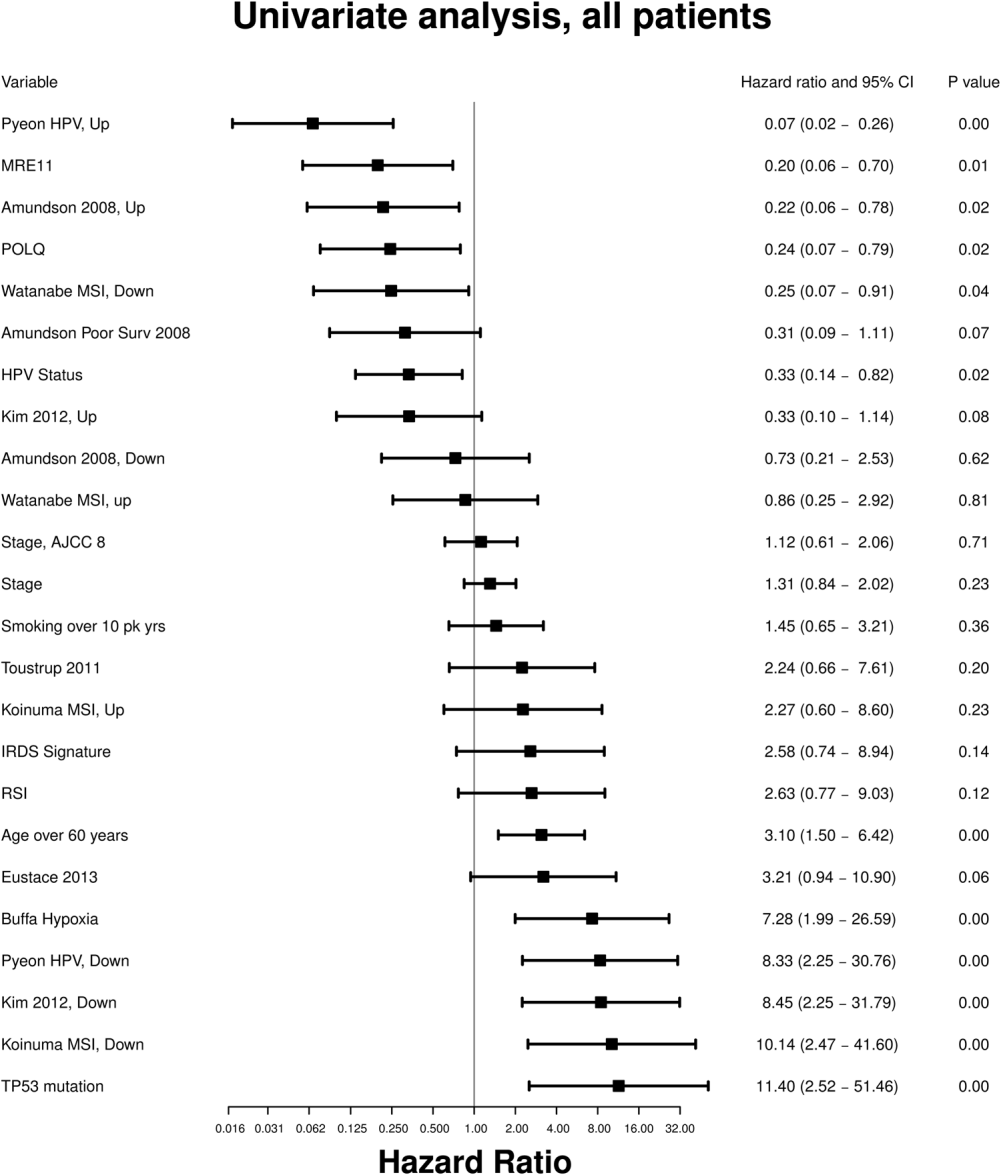
Hazard ratios for overall survival in univariate predictor model for each gene signature and clinical covariate considered.

We next examined the prognostic ability of each signature in univariate analysis of overall survival (Fig. 4), both in the series as a whole and in the two subgroups of HPV+ and HPV− tumors.

### Hypoxia gene signatures

One signature of hypoxia (Buffa^25^) was significant on univariate analysis (HR = 7.28, 95% CI 1.99–26.59, p < 0.01), but not once the series was divided into HPV+ and − subgroups, or on multivariate analysis.

### Microsatellite instability signatures

The signature Watanabe MSI, Down^32^ was significant for the whole series (HR = 0.25, 95% CI 0.07–0.91, p = 0.04). In the HPV-ve subgroup, the Watanabe MSI, Down^32^ (HR = 0.25, 95% CI 0.07–0.93, p = 0.04) and the Koinuma MSI, Down^31^ (HR = 5.72, 95% CI 1.27–25.83, p = 0.02) gene signatures were significant predictors of survival. In multivariate analysis of the whole series, the Koinuma MSI, Up^31^ (HR = 6.99, 95% CI 1.73–28.23, p = 0.01) and Koinuma MSI, Down^31^ (HR = 10.24, 95% CI 1.65–63.67, p = 0.01) gene signatures were both significant predictors of poorer survival. In multivariate analysis of HPV− patients, Koinuma MSI, Up^31^ (HR = 6.33, 95% CI 1.31–30.66, p = 0.02) and Koinuma MSI, Down^31^ (HR = 11.82, 95% CI 1.95–71.46, p = 0.01) gene signatures were significantly predictive of poorer survival.

### Radiosensitivity gene signatures

Two signatures of radiosensitivity: Amundson, Up^23^ (HR 0.22, 95% CI 0.06–0.78, p = 0.02) and Kim, Down^24^ (HR 8.45, 95% CI 2.25–31.79, p < 0.01) were significant in the whole series, but not in subgroup analysis, or multivariate analysis.

### Gene signature for HPV status

In univariate analysis, Pyeon HPV, Down^41^ was a significant predictor of survival across the series as a whole (HR 8.33, 95% CI 2.25–30.76, p < 0.01). Among HPV+ tumors, the Pyeon HPV, Up^41^ gene signature showed statistical significance (HR = 0.02, 95% CI < 0.01–0.86, p = 0.04) as a positive predictor of survival, and the Pyeon HPV, Down^41^ gene signature (HR = 362.4, 95% CI 1.86–70558, p = 0.03) was a predictor of poorer survival. Surprisingly, among HPV− tumors, the Pyeon HPV, Up^41^ (HR = 0.15, 95% CI 0.03–0.62, p = 0.01) gene signature still showed significant positive predictive value. In multivariate analysis of the whole series, the Pyeon HPV, Up^41^ (HR = 0.09, 95% CI 0.02–0.51, p = 0.01) signature remained significantly positively predictive of survival. In multivariate analysis of HPV− patients, Pyeon HPV, Up^41^ gene signature was again also significantly predictive of better survival (HR = 0.09, 95% CI 0.02–0.47, p < 0.01).

### Individual genes

The single genes MRE11 and POLQ, and TP53 mutation status, were significant univariate predictors of survival. We found a highly significant non-random association of non-silent TP53 mutation to HPV negativity (Fisher’s exact test Odds Ratio = 0.14, p < 10^−9^), suggesting an underlying association, and this was not included in the multivariate analysis. Furthermore, TP53 mutation was a strong univariate negative predictor of survival when considered across all patients (HR = 11.40, 95% CI 2.52–51.46, p < 0.01).

### Clinical factors

Finally, the clinical variables of age greater than 60 years (HR = 3.10, 95% CI 1.50–6.42, p < 0.01) and HPV status (HR = 0.33, 95% CI 0.14–0.82, p = 0.02) were also significant across the whole series. The administration of radiotherapy was not a significant predictor of survival in the overall series (HR = 0.67, 95% CI 0.33–1.36, p = 0.27). AJCC 7 Stage was not significant in univariate analysis (Fig. 4, HR 1.31, 95% CI 0.84–2.02 p = 0.23). In the HPV-ve series, age > 60 was a strong negative predictor of survival (HR = 2.62, 95% CI 1.13–6.09, p = 0.02); age > 60 was also a strong predictor in multivariate models with all signatures except Watanabe MSI, Down^32^ (HR = 2.41, 95% CI 0.92–6.33, p = 0.07). For all other signatures, age > 60 was statistically significant in multivariate analyses with HR 2.67–3.66, CI 1.05–9.43, p < 0.05. Overall, a number of signatures (including those related to hypoxia, MSI and radiosensitivity) trended towards significance upon subgroup analysis, but analysis suffered from reduced sample size. No gene signatures remain statistically significant predictors of survival for the HPV+ tumor group in multivariate analysis (though this was limited by sample size).

### Integration of gene signatures and exploration of their correlation

**Figure 5:**
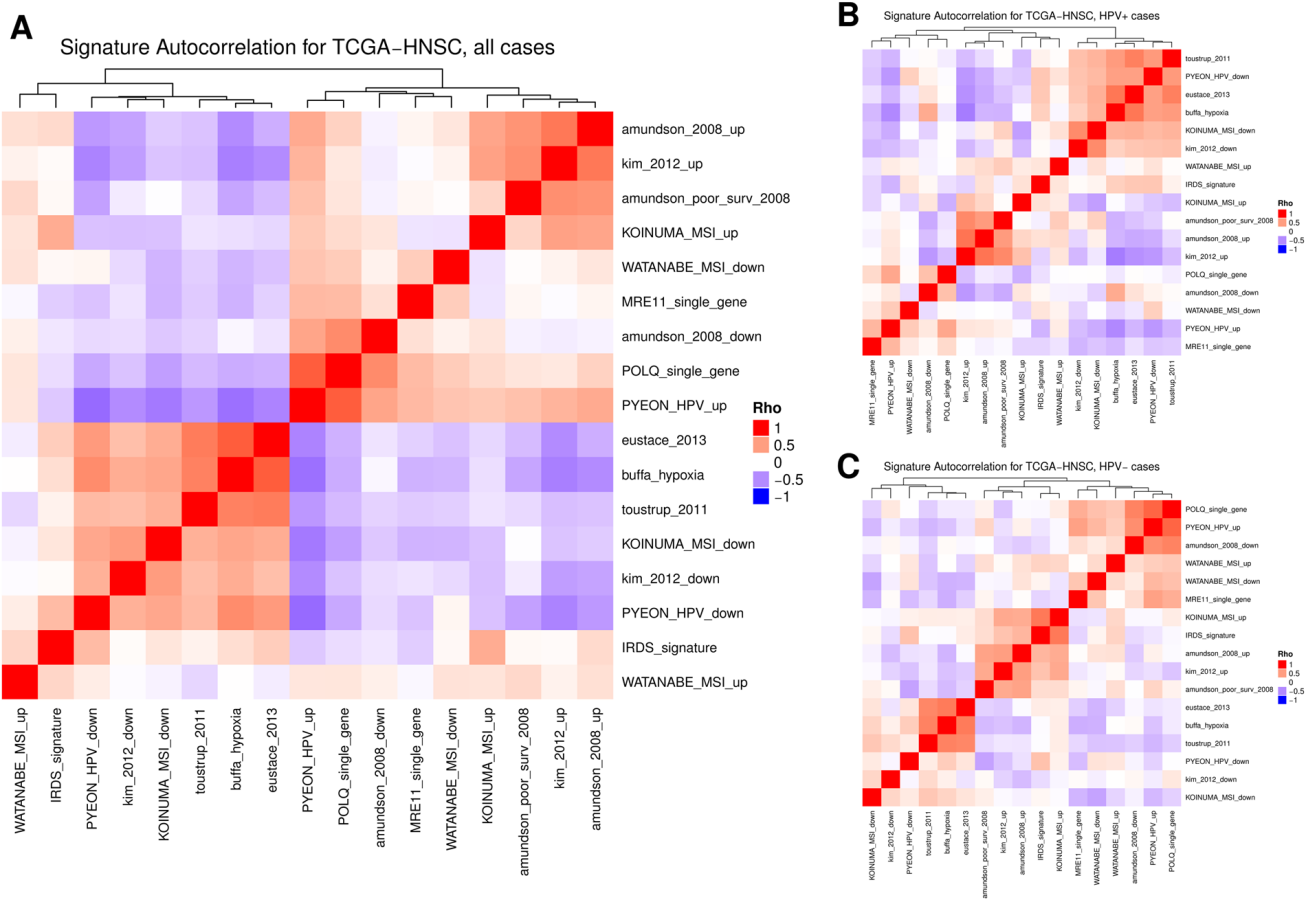
Co-correlations of gene signature scores among oropharynx cancers (**A**), and within HPV positive (**B**) oropharynx cancers, and HPV negative (**C**) oropharynx cancers. Gene signatures cluster into two groups when considered among all oropharynx cancers, but clustering shows differences when samples stratified by HPV status.

Following this, we assessed the Spearman correlation between the median of signature gene expression in each sample to determine whether the gene signatures captured similar information across patient samples. Statistical significance in the form of p-values is provided for each of these correlations in Appendix B. As shown in Fig. 5, depicting the heatmap of correlation coefficients, there are two highly clustered groups of gene signatures; likely due to similar sets of genes capturing consistent biology. One cluster contains the signatures by Kim^24^, Toustrup^27^, Eustace^26^, Buffa^25^, and has genes downregulated in MSI and HPV, associated with hypoxia, and the second cluster contains gene signatures by Amundson^23^, Kim (up)^24^, MSI up^32^, and HPV up^41^, on the opposing side. The overlap of the specific genes themselves between the various signatures is relatively low, with there being 4–12 genes shared between the Toustrup^27^ (16 genes), Eustace^26^ (23 genes), and Buffa^25^ hypoxia signatures (53 genes). The Kim^24^ (30 genes) and Amundson survival^23^ (168 genes) signatures also overlapped by 4–8 genes. A full plot of the overlaps of the genes used in each signature is available in Supplementary Figure 2. Interestingly, when stratified into the HPV+ and HPV− subgroups, we observed stark differences in the way the gene signatures correlated with one another. For HPV+ there was more consensus among hypoxia-mediated signatures and the RSI^22^ while for the HPV−, there was a greater degree of consensus among MSI and immune based signatures.

## Discussion

In this work, we attempted to establish what ‘additional value’ gene signatures (of radiation response and tumour biology) add to the accepted clinico-pathological variables which are currently used to determine treatment in OPC. First, we showed that certain gene signatures and TP53 mutation status are strong univariate predictors of prognosis in OPC. Second, by performing subgroup analyses for predictive value in HPV+ and HPV− subgroups, we revealed differences in the prognostic ability of gene signatures between these groups. Interestingly, the Pyeon^41^ HPV signature showed strong prognostic ability across subgroups, including HPV−, suggesting that this signature may capture heterogeneity beyond the binary classification afforded by clinical HPV status. Multiple genes in the signature capture cell cycle deregulation, agreeing with emerging data that cell cycle dysregulation is a mechanism of radioresistance in HPV-ve HNSCC^42^. This hypothesis could be investigated further in future biomarker-driven studies, particularly combined with emerging sequencing data.

Recent clinical studies^43^ show differing biological behaviour of OPC suggestive of underlying biological characteristics^44^. This supports our hypothesis that the strong prognostic value of the HPV and MSI signatures in multivariate analysis reflects inter-tumour heterogeneity beyond HPV status as a binary variable (particularly cell cycle and genome instability genes which are represented in the signatures)^41^. TP53 status has been shown in many studies, including ours, to be a powerful predictor of outcome but is not currently assessed in routine clinical practice. The clinical focus of current research in OPC is on de-escalation of treatment, although clinical trials have shown contradictory results. Our results confirm that personalisation of treatment for HPV+ patients, particularly in those who have additional mutations such as TP53 (often associated with smoking, underlying the use of smoking as a surrogate marker), needs to be performed in a clinical trial^9^.

More generally, this study also provides perspective on the clinical role of gene signatures, as these specialised tests become more widely available. We highlight multiple issues including reproducibility. These gene signatures ostensibly have biologic relevance and were validated on the datasets they were derived on, but showed significant differences in behaviour and quality when tested on an independent, clinical dataset. Moreover, we emphasise the importance of reproducible metrics for gene signatures. The method of signature scoring in each sample is as important as the signature components themselves. *sigQC*^40^ aims to alleviate this issue by testing multiple different scoring metrics, and then comparing a rank correlation between them, thereby testing reproducibility of ordering of samples with respect to signature scores (important during both signature derivation and validation phases).

Differences in signature characteristics that may lead to poor reproducibility are numerous; for instance, the manner in which the signature was derived, due to inter-platform differences (e.g. microarray vs. RNA-seq), or batch effects. As a result, these signatures lack the ability to be validated across cohorts without the use of a targeted, prospective clinical study, limiting wider adoption, and suggest that quality testing with tools such as *sigQC* is of importance during signature derivation, particularly when used for iterative refinement of signatures from a candidate signature, to determine whether reproducibility can be enhanced. Indeed, some of the considered signatures that we have included in our analysis had the risk of being too narrowly defined to be applied to a more heterogeneous population than they were originally defined for. In this manuscript, we attempt to also shed light on these discrepancies and in our analysis have assessed signature quality using *sigQC*. Nevertheless, issues of generality are still possible, and in this instance, signature gene expression may not be entirely representative of the process of interest in the expanded population of samples.

Our study is limited by patient numbers, the retrospective nature of TCGA data, and by only being able to investigate in one series. Moreover, HPV status was determined by p16 IHC in some studies and by HPV ISH, and there is known to be minor differences in sensitivity and specificity between these methods, beyond the scope of this paper. In addition, while TCGA is unusually complete for a single series, it does not report performance status, and suffers bias such as the high proportion of smokers. The radiotherapy treatment information was very limited as to whether adjuvant or definitive. Prospective validation of existing (or indeed novel) gene signatures of outcome in OPC^45^, in a series treated by protocol, staged with the 8^th^ edition of the staging system, will be essential for widespread clinical adoption of gene signatures.

## Conclusion

Several gene signatures representing HPV and microsatellite instability remained significant on multivariate analysis, suggesting significant heterogeneity exists in OPC, beyond the dichotomy of HPV status. We found gene expression signatures suggested hypotheses of underlying biology, but quality control and independent validation limit their current value above accepted clinico-pathological variables.

## Supplementary information

Supplementary Information.
