# Supplementary material for: Role of gene signatures combined with pathology in classification of oropharynx head and neck cancer
Source: Sci Rep. 2020 Jun 23;10:10226. doi: 10.1038/s41598-020-66983-x (PMC7311543; doi:10.1038/s41598-020-66983-x)
Supplement: Supplementary file 1 — Supplementary Information. [file 41598_2020_66983_MOESM1_ESM.pdf]

Supplementary Information:

Title:

'Role of gene signatures combined with pathology in classification of oropharynx head and neck cancer'

Authors:

Andrew Dhawan[1,*], MD, DPhil, Jacob Scott[2], MD, DPhil, Purnima Sundaresan[3,4], MBBS, PhD, FRANZCR, Michael Veness[3,4], MMed, MD, FRANZCR, Sandro Porceddu[5,6], MBBS, MD, FRANZCR, Eric Hau[3,4], MBBS, PhD, FRANZCR, Adrian L. Harris[1], MBChB, DPhil, Francesca M. Buffa[1], PhD, Harriet E. Gee[3,4,*], MBBS, DPhil, FRANZCR

Affiliations:

[1]Department of Oncology, University of Oxford, Oxford, United Kingdom

[2]Translational Hematology and Oncology Research, Cleveland Clinic, Cleveland, Ohio, USA

[3]Crown Princess Mary Cancer Centre, Westmead Hospital, Westmead, New South Wales, Australia.

[4]Sydney Medical School, C24 - Westmead Hospital, The University of Sydney, Sydney, New South Wales, Australia

[5]Princess Alexandra Hospital, Woolloongabba, Queensland, Australia

[6]Faculty of Medicine, The University of Queensland, Brisbane, Queensland, Australia

Appendix A:

Supplementary Table A1: Details of series

SERIES 1: TCGA:

Characteristic	HPV positive (N=42)	HPV negative (N=72)
Median age in years at diagnosis (IQR)	53.5 (49.25 - 58.75)	59 (52.75 - 67.00)
Number female (%)	5 (11.9%)	17 (23.6%)
Subsite:		
Base of Tongue	10 (24%)	29 (40%)
Oropharynx	2 (5%)	23 (32.3%)
Tonsil	30 (71%)	20 (27.7%)
Centrally assigned stage (AJCC 7):		
I	3 (7%)	7 (10%)
II	5 (12%)	11 (15%)
III	5 (12%)	13 (18%)
IV	29 (69%)	41 (57%)
Method of HPV status determination[1]		
HPV RNA positive (RNA seq)	42 (100%)	0 (0%)
HPV DNA (ISH) positive	19 (45%)	0 (0%)
p16 immunohistochemistry (IHC) positive	31 (73%)	2 (2%)
Unknown ISH/IHC	11 (26%)	32 (46%)
Number with more than 10 pack year history (%)	17 (40.4%)	42 (58.3%)
Median pack years of smokers (IQR)	25.5 (12 - 37.5)	40 (29.75 - 60)
Number with non-silent TP53 mutation[2] (%)	4/19 (21.1%)	20/28 (71.4%)
Number of patients receiving radiotherapy (%)	26 (62%)	44 (61%)
Number of patients receiving surgery (%)	22 (52%)	63 (88%)

Number of patients receiving chemotherapy (%)	10	4
<u>'Method of curative treatment'</u>[3]		
Concurrent chemoradiotherapy	9	3
Radiation alone	2	2
Surgery alone	12	3
Sequential multimodality therapy	1	1
Not stated	18	63
Median follow up (years)	1.9	1.7
Number of events (overall survival)	10	12
Number of events (recurrence free survival)	11	13

[1] Samples were classified as HPV-positive as described in (reference Comprehensive genomic characterization of head and neck squamous cell carcinomas The Cancer Genome Atlas Network Nature volume 517, pages576–582(2015) using an empiric definition of >1,000 mapped RNA sequencing (RNA-Seq) reads, primarily aligning to viral genes E6 and E7. Methods used by TCGA for p16 staining, ISH or RNAseq are further described in Supplementary File 2, section S1.2. In particular, p16 immunohistochemistry was scored in binary fashion as positive when strong homogeneous cytoplasmic and/or nuclear staining was found and negative if no staining or focal heterogeneous nuclear or cytoplasmic staining was observed.

[2] Note that denominator is different when considering non-silent TP53 mutated cases because cases are further subsetted to samples for which mutational data is available.

[3]Column in TGCA referring to primary method of curative treatment is missing data for most patients

SERIES 2: Wichmann:

Characteristic	Series overall for AJCC 7 (n=101)	Series overall for AJCC 8 (n=100)	HPV positive (n=54)	HPV negative (n=46)
Median age in years at diagnosis (range)	58 (53-64)	58 (52–62)	59 (45-79)	58 (44-78)
Number female (%)	18 (18%)	18 (18%)	13 (24%)	5 (11%)
Subsite:				
Base of tongue			0	0
Oropharynx			54 (100%)	46 (100%)
Tonsil			0	0
Number with more than 10 pack year history (%)	78 (77%)	79 (79%)	36 (67%)	43 (93%)
HPV detection method[1]				
DNA only	25 (25%)	25 (25%)	25 (46%)	0 (0%)
RNA and DNA	29 (29%)	29 (29%)	29 (54%)	0 (0%)
p16	N/A	N/A	N/A	N/A
Median pack years of smokers (IQR)	30 (10-40)	30 (12 - 40)	15 (0-40)	35 (28-40)
Number with non-silent TP53 mutation (%)	13/73 (18%)	13/73 (18%)	5/41 (12%)	7/32 (22%)
Number of patients receiving radiotherapy (%)	101 (100%)	100 (100%)	54 (100%)	46 (100%)
Number of patients receiving surgery (%)	0	0	0	0

Number of patients receiving chemoradiotherapy (%)	74 (73%)	74 (74%)	38 (70%)	35 (76%)
Median follow up (years)	2.55	2.57	2.70	2.42
Number of events (overall survival)	36	35	19	17
Number of events (recurrence free survival)	50	49	26	27

[1]HPV status was considered positive if test was positive for HPV DNA, or DNA and RNA. No samples were positive for RNA but not DNA. As described in the paper (Wichmann ref), 'the HPV 16 DNA status and genotype were determined in 100 ng DNA of each sample using the INNO-LiPA HPV Genotyping Extra (Innogenetics, Gent, Belgium). RNA samples of HNSCC positive for the subtype HPV16 underwent analysis of E6*I transcripts by RT-PCR as previously described. A sample was concluded to be HPV16 RNA+ve whenever HPV16 E6*I transcripts were detected'. p16 immunohistochemistry was not performed.

SERIES 3: Walter:

Characteristic	Series overall for AJCC 7 (n=34)	Series overall for AJCC 8 (n=24)	HPV positive (n=10)*	HPV negative (n=14)*
Median age in years at diagnosis (range)	57 (47-63)	58 (44-79)	59 (45-79)	58 (44-78)
Number female (%)	9 (26%)	6 (25%)	3 (30%)	3 (21%)
Subsite:				
Base of tongue			0	0
Oropharynx			10 (100%)	14 (100%)
Tonsil			0	0
Number with more than 10 pack year history (%)	26 (76%)	15 (63%)	4 (40%)	11 (79%)
Median pack years of smokers (IQR)	20 (3-37)	20 (0-40)	3 (0-20)	30 (9 - 49)
Number with non-silent TP53 mutation (%)	N/A	N/A	N/A	N/A
Number of patients receiving radiotherapy (%)	29 (85%)	21 (88%)	10 (100%)	11 (79%)
Number of patients receiving surgery (%)	14 (41%)	12 (50%)	5 (50%)	7 (50%)
Number of patients receiving chemotherapy (%)	25 (75%)	19 (80%)	9 (90%)	10 (71%)
Median follow up (years)	3.2	3.2	4.75	1.67

Number of events (overall survival)	22	15	5	10
Number of events (recurrence prior to death)	13	9	3	6

*HPV status was determined using in situ hybridisation for DNA of high risk genotypes as described in (36); 10 patients had unknown HPV status and were excluded from further analysis.

SERIES 4: Gee:

Characteristic	Series overall (n=21)	HPV positive* (n=10)	HPV negative (n=11)
Median age in years at diagnosis (IQR)	60 (54-64)	54 (51-56)	63 (60-72)
Number female (%)	4 (19%)	2 (20%)	2 (18%)
Subsite:			
Base of tongue	10 (48%)	4 (40%)	6 (54%)
Oropharynx	4 (19%)	1 (10%)	3 (27%)
Tonsil	7 (33%)	5 (50%)	2 (19%)
Number with more than 10 pack year history (%)	14 (67%)	7 (70%)	7 (64%)
Median pack years of smokers (IQR)	N/A	N/A	N/A
Number with non-silent TP53 mutation (%)	N/A	N/A	N/A
Number of patients receiving radiotherapy (%)	21 (100%)	10 (100%)	11 (100%)
Number of patients receiving surgery (%)	21 (100%)	10 (100%)	11 (100%)
Number of patients receiving chemotherapy (%)	1 (5%)	1 (10%)	0 (0%)
Median follow up (years)	3.6	4	3.6
Number of events (overall survival)	3	1	2
Number of events (recurrence free survival)	3	1	2

*HPV status determined via p16 immunohistochemistry as described in (ref TCGA), p16 was scored in binary fashion as positive when strong homogeneous cytoplasmic and/or nuclear staining was found and negative if no staining or focal heterogeneous nuclear or cytoplasmic staining was observed. HPV status by DNA/RNA was not performed.

Supplementary Table A2: Listing of gene signatures considered in this study and associated references.

Gene signature name	Number of genes	Biological parameter measured	Method of derivation and platform	Reference
Amundson 2008, Down	5	Radiation sensitivity	NCI-60 cell lines, microarray	(23)
Amundson 2008, Up	16	Radiation sensitivity	NCI-60 cell lines, microarray	(23)
Amundson 2008, Poor survival	161	Radiation sensitivity	NCI-60 cell lines, microarray	(23)
Radiation Sensitivity Index (RSI)*	10	Radiation sensitivity	Cell lines, RNA microarray, linear regression analysis	(40)
Kim 2012, Down	20	Radiation sensitivity	NCI-60 cell lines, 4 RNA microarray platforms	(24)
Kim 2012, Up	10	Radiation sensitivity	NCI-60 cell lines, 4 RNA	(24)

			microarray platforms	
Buffa, Hypoxia	53	Hypoxia	Clinical datasets, microarray platforms	(25)
Eustace 2013	23	Hypoxia	Clinical samples, TaqMan low density array PCR cards	(26)
Toustrup 2011	14	Hypoxia	Cell lines, xenografts and clinical samples, microarray	(27)
Pyeon, HPV, Down	10	HPV	Clinical samples, microarray	(39)
Pyeon, HPV, Up	86	HPV	Clinical samples, microarray	(39)
IRDS signature	38	Immune response	Cell lines, microarray	(28)
Koinuma, MSI, Down	8	MSI/Immune response	Clinical samples, microarray	(31)

Koinuma, MSI, Up	18	MSI/ Immune response	Clinical samples, microarray	(31)
Watanabe, MSI, Down	76	MSI/ Immune response	Clinical samples, microarray	(32)
Watanabe, MSI, Up	27	MSI/ Immune response	Clinical samples, microarray	(32)

* RSI signature was included along with associated linear model. Other signatures did not have published risk models, and for these we used the median expression of signature genes as the signature score.

Supplementary Table A3: Single genes considered and the references supporting their inclusion as radiosensitivity predictors.

Gene name	Reference
MRE11	(41)
POLQ	(18,42)

Supplementary Table A4 – overlap between methods of HPV status determination for 'Combined cohort' HPV positive cases. For details of methods please see Supplementary Table A1 and individual references.

**Method**	**Positive**	**Negative**	**Unknown**
**p16 IHC**	10	24	82
**HPVDNA**	83	0	31
**HPVRNA (RNAseq or RNA RT-PCR)**	71	25	20

Supplementary Figure 1 Flow chart for the selection/search for gene expression signatures. HPV = human papilloma virus, TCGA = The Cancer Genome Atlas, MSigDB – details at https://www.gsea-msigdb.org/gsea/msigdb/index.jsp

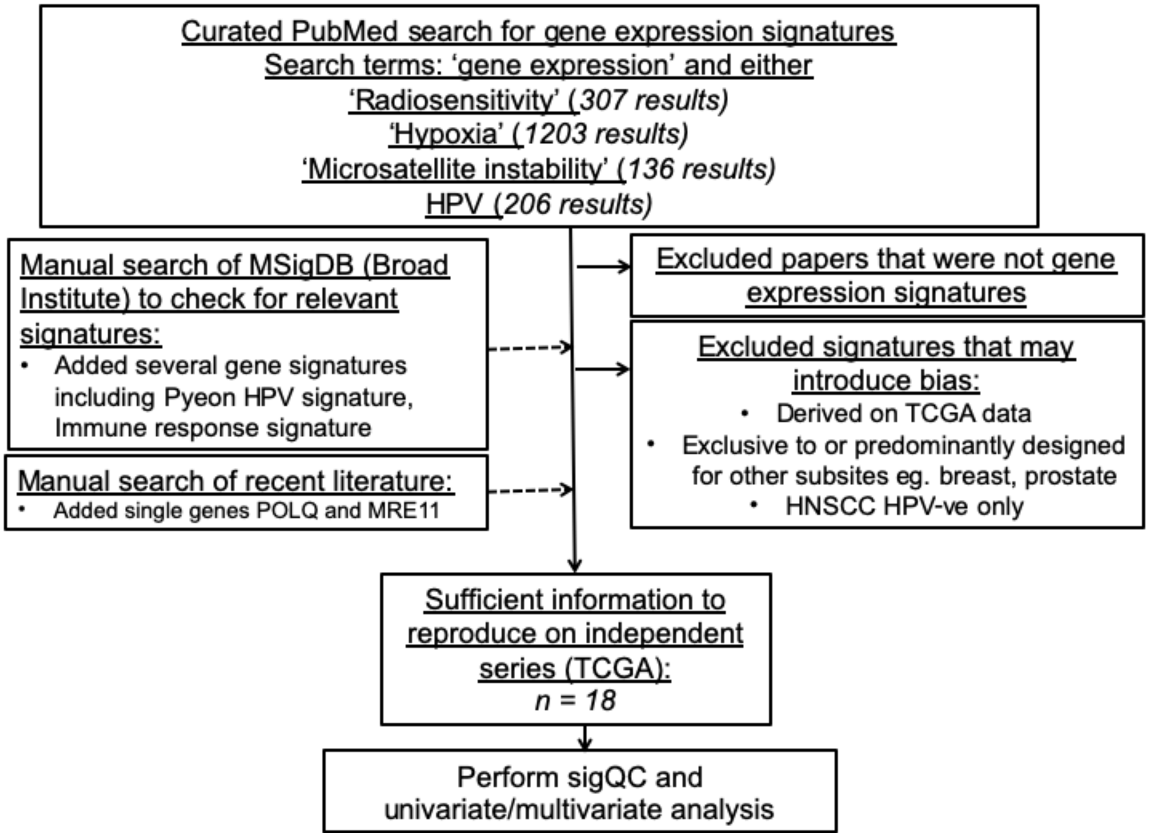

Appendix B: Supplementary Files

- Listing of all gene signatures
- sigQC results for all signatures
- Univariate analyses for survival in HPV positive and negative subgroups
- Multivariate analyses for survival in all patients, HPV positive, and negative groups, for the gene signature score

These files are available for download at:
https://doi.org/10.5281/zenodo.3669128
